# Industry Collaborations of Research Teams: Are They Penalized or Rewarded in the Grant Evaluation Process?

**DOI:** 10.3389/frma.2021.707278

**Published:** 2021-10-21

**Authors:** Sıla Öcalan-Özel, Patrick Llerena

**Affiliations:** BETA, Université de Strasbourg, Strasbourg, France

**Keywords:** industry collaboration, diversity, prior experience, grant peer review, research funding

## Abstract

This paper explores the relationship between the industry collaborations of grant applicant teams and the outcomes of a multistage grant evaluation process. We studied this relationship by focusing on two possible channels of impact of industry engagement—team diversity (or the diversity effect) and prior collaboration experience (or the experience effect)—and examined their influence on the evaluators' decision by using the proxies of direct industry engagement (i.e., the involvement of a company-affiliated researcher in the grant applicant team) and indirect industry engagement (i.e., joint publications with a company-affiliated researcher prior to the grant application), respectively. We analyzed data extracted from the application and reviewed materials of a multidisciplinary, pan-European research funding scheme—European Collaborative Research (EUROCORES)—for the period 2002–2010 and conducted an empirical investigation of its three consecutive grant evaluation stages at the team level. We found that teams presenting an indirect engagement were more likely to pass the first stage of selection, whereas no significant relationships were found at any of the three evaluation stages for teams presenting a direct engagement. Our findings point to the heterogeneity of the decision-making process within a multistage grant evaluation scheme and suggest that the policy objective of fostering university–industry collaboration does not significantly impact the funding process.

## Introduction

With the emergence of knowledge-based economy, promoting university–industry (U–I) knowledge and technology transfer (KTT) has become one of the main objectives of science, technology, and innovation (STI) policies (Geuna and Muscio, 2009). To achieve this goal of stimulating KTT, a recently published OECD report (2019) on U–I collaboration shows that the organization's member states deploy different policy mixes, combining financial, regulatory, and soft policy instruments. One of the policy tools that has contributed most to the recent evolution of the institutional framework underpinning the STI policy has been research funding, in the form of both block and competitive grants. However, while the latter are among the most widely used financial instruments across OECD countries, there are marked heterogeneities in terms of the budgets allocated, the duration of these grants, their beneficiaries, and the selection criteria employed (OECD, 2019).

Funding agencies around the world award grants for research, some of which are dedicated directly to promoting U–I collaboration. Such direct collaborative grant schemes aim principally at enhancing business innovation and boosting the social return from public investment in science and international competitiveness (Cunningham and Gök, 2012); at the same time, they are also becoming common in both low- and middle-income countries, as they strive to catch up with their more developed counterparts (Bruhn and McKenzie, 2019). Yet, even in the cases in which a grant program is not explicitly aimed at promoting KTT, those involved in the decision-making process may be inclined to foster U–I collaboration (Banal-Estañol et al., 2019a). Thus, in practice, evaluators who look favorably (or unfavorably) on U–I KTT might be more (or less) likely to take into consideration the industry collaboration activities of an applicant team when allocating public funds.

In this study, we seek to determine whether the industry engagements of applicant teams are penalized or rewarded by evaluators at the various stages of the grant evaluation process. In practice, industry collaborations may be penalized because evaluators can deem them to be of little or no value for scientific output, or they may simply wish to avoid the risk that the collaborative firm will appropriate the publicly funded research result. This latter argument finds some support, as it has been shown that the propensity of academic scientists to share information tends to be lower when there is a risk of appropriation (Haeussler, 2011). Unlike academic science, where conformity to the norms of openness (Merton, 1973) is the widely accepted practice, industrial science is often guided by secrecy given the commercial interests involved (Cohen et al., 2000), resulting in a lower propensity for information-sharing among such scientists. For these reasons, evaluators might penalize industry collaboration.

In contrast, some evaluators might show a greater propensity to favor applicant teams' industry collaborations, especially in certain application-oriented disciplines such as engineering. Funding agencies and evaluators that seek to foster U–I collaboration may opt to award grants to teams that present a clear industry engagement. Thus, they are likely to select applicant teams that show themselves capable of producing either technologies that have the potential for industrial application or original research that can permit the transfer of knowledge, or teams that have previous collaboration experience that has resulted in the development of KTT networks. To the best of our knowledge, there has been no attempt to date to examine the relationship between U–I collaborations and the grant allocation process, with the exception of Banal-Estañol et al.'s (2019a). Yet, even here, the authors do not directly address this specific relationship, and they do not seek to offer an in-depth explanation of the forces at play. Indeed, the study limits itself to the disciplines of the physical sciences and engineering in the United Kingdom and concerns itself solely with the final outcome of the grant application process (i.e., successful or otherwise).

Here, to account for the potential effects of industry collaboration on the research funding process, we examine the role of two possible channels of impact of the U–I collaborations of applicant teams during the grant allocation process. The first is given the label *diversity effect* and is captured by the *direct* industry engagement of applicant teams, and the second is labelled the *experience effect* and is captured by the *indirect* industry engagement of applicant teams. By using *direct* and *indirect* industry engagements as proxies, we are able to measure the impact of team diversity and prior experience of industry collaboration on the decisions made by different evaluators (namely, the members of an Expert Panel and External Reviewers) in the multistage, multidisciplinary, pan-European grant scheme of EUROCORES (European Collaborative Research) managed by the European Science Foundation (ESF).

We examine the nature of *direct* industry engagement in the light of discussions in the diversity literature, where diverse teams have been shown to more likely exhibit both higher ex-post performance (Williams and O’Reilly, 1998) and produce novel combinations and breakthrough research (Arts and Veugelers, 2015). In this respect, we conjecture that applicant teams having an industry/company-affiliated partner in the grant application are more likely to produce original research, as they are diverse in knowledge and skills, and more likely to produce transferable technology for the industry given that they are in direct proximity to the industry network. We refer to this as *direct* industry engagement or the *diversity effect*.

Additionally, evaluators may select applicant teams on the grounds that they already present certain embedded characteristics, including prior experience of industry collaboration for the transfer of knowledge and technology. In this respect, we conjecture that applicant teams with past experience of industry engagement should be more capable of KTT in the future. As a proxy of prior experience, we consider applicant teams that include a member with a joint-publication with industry/company prior to the present grant application. We refer to this as *indirect* industry engagement or the *experience effect*.

To test these two conjectures concerning the relationships between *direct/indirect* industry engagement and the outcomes of the three consecutive stages in a grant evaluation process, we draw on a sample, providing information about 10,533 EUROCORES grant applicants across all scientific domains, corresponding to 1,642 outline proposals and 886 full proposals for the period 2002–2010. The empirical results suggest that teams with an *indirect* industry engagement are more likely to pass the first stage of selection (outline proposals) conducted by Expert Panel members, whereas no significant link can be established for the subsequent evaluation stages (full proposals) conducted, first by External Reviewers and second by Expert Panel members. More specifically, we are unable to observe a significant link between *direct* industry engagement and the outcome of the grant evaluation process at any of its stages. The presence (or otherwise) of such a correlation may, among other reasons, be attributable to the heterogeneity in the decision-making processes perpetuated by different actors at subsequent stages (e.g., collective vs individual decision making; decision-making subject to time constraints; and having to access detailed information) or to the objective of the funding agency that explicitly recognizes the wish to promote U–I collaboration. In the case of EUROCORES, the explicit objective of the grant scheme was to stimulate the highest quality innovative, multidisciplinary, and investigator-driven collaborative research, by bringing together different scientists and national research funding organizations; however, fostering U–I collaboration was not an explicitly stated aim.^1^ Nonetheless, as Banal-Estañol et al. (2019a) argue, even in the absence of such an explicit aim, the evaluators may have been implicitly inclined to foster industry collaborations.

The rest of the paper is structured as follows. The *Grant Evaluation Process and Industry Engagement* section presents a general framework in which the grant peer-review process is outlined, the *diversity* and *experience* effects and their respective proxies of *direct* and *indirect* industry engagements are described, and the EUROCORES funding scheme is explained. The *Data and Methodology* section describes the data, variables, and the methodology used. The *Econometric Results* section presents the empirical results for the three consecutive evaluation stages, and the *Discussion and Conclusion* section concludes by discussing these outcomes.

## Grant Evaluation Process and Industry Engagement

### Grant Peer-Review Process

Although some funding agencies award grants on the basis of their own in-house evaluations, a major proportion of competitive grants are allocated using a peer-review process (Stephan, 2012; Roumbanis, 2019). This can have both positive and negative outcomes: on the one hand, a peer-review system encourages researchers to remain productive throughout their careers, on the other hand, the system discourages risk taking, tending to support projects assessed as having a high degree of “doability” (Stephan, 2012), and thus the peer-review process has a marked influence on the direction and orientation of scientific research in general.

Funding organizations typically state that they allocate grants in accordance with a project's scientific merit or excellence. Indeed, there is clearly a need to allocate scarce resources to feasible research projects with the potential to generate high-quality output of notable impact (Tijssen et al., 2002). However, claims have been made that funding decisions are not simply based on scientific merit or excellence but that they are also influenced by, among other factors, the personal attributes of individual applicants (Witteman et al., 2019), the structure of the research teams (Banal-Estañol et al., 2019b), the personal attributes of the peer reviewers evaluating the projects (Gallo et al., 2016), the intellectual distance (Boudreau et al., 2016), and the network ties between the applicants and their evaluators (Ebadi and Schiffauerova, 2015).

The grant peer-review process appears to be prone to different types of bias, being influenced by factors unrelated to a project's actual quality (see, for example, Wennerås and Wold, 1997, for a discussion of the sexism inherent to the process). Such bias might be both intentional and unintentional, given that the funding process is characterized by a whole series of uncertainties regarding the true quality of a research proposal. A review of the literature reveals the different types of bias with which applicants have to contend. Bromham et al. (2016), for instance, found that high levels of interdisciplinarity reduce the likelihood of attracting funds. The authors claim that while interdisciplinary research is often encouraged at the policy level, funding organizations or reviewers tend to favor more focused research, pointing to what has been called an “interdisciplinarity paradox” (Woelert and Millar, 2013). This interdisciplinarity bias may reflect the fact that evaluators have a highly focused expertise, and hence find it difficult to evaluate all sections of a given project (Woelert and Millar, 2013; Bromham et al., 2016). A similar bias may occur in reaction to novelty. Indeed, novel projects are difficult to evaluate as they involve a high degree of uncertainty and are thus usually perceived as riskier in terms of feasibility (Boudreau et al., 2016; Stephan et al., 2017; Wang et al., 2017).

In this study, among the various factors that might affect a funding decision, we are specifically concerned with the impact of industry engagements or collaborations of research teams on the grant evaluation process. In particular, we examine the effects of a team's *direct* and *indirect* industry engagements in terms of the *diversity effect* of the former and the *experience effect* of the latter.

### Structural Diversity and Experience of Prior Collaborations

In this subsection, we discuss the impact of the industry engagement of research teams on the grant allocation process in relation to two factors: team diversity and prior experience. In a grant allocation process, KTT policy goals can be achieved either by selecting researchers who are able to produce original research outcomes with a high potential for transfer to industry or by selecting researchers with prior experience of KTT and who are, therefore, more likely to continue to foster transfer in the future.

The research performance of teams has been associated with their diversity (Horwitz, 2005; van Knippenberg and Mell, 2016); thus, teams that can draw on different knowledge bases and skills are more likely to produce new combinations and more breakthrough research (Arts and Veugelers, 2015). It should be noted, however, that *diversity* may be used to refer to different attributes of a team. Indeed, the distinction between demographic and structural diversity has been highlighted in this body of literature (see Williams and O’Reilly, 1998; Cummings, 2004), with the former referring to easily observable characteristics, such as gender, age, and race, and the latter referring to cognitive abilities. Diversity in cognitive attributes is a widely accepted condition leading to novel combinations and hence to radical innovations in the Schumpeterian sense (Nelson and Winter, 1982; Fleming, 2007). Cummings (2004) shows that structurally diverse teams—comprising members with different organizational affiliations, roles, or positions and with distinct knowledge bases and skills—present performance benefits, especially when these members share knowledge outside of the group. Hence, teaming up with an external partner contributes to structural diversity and increases the potential to produce original or novel research.

The potential for producing original research might be a factor favored by evaluators in the grant peer-review process. By positively considering the structural diversity introduced by a project's industry partners, evaluators can target two objectives: they can increase team diversity and can facilitate KTT to the industry. Yet, too much diversity can increase communication and coordination costs between team members (Dahlin et al., 2005). Moreover, the novel methods and theories that are likely to be developed by diverse teams—in particular, those considered frontier research—are subject to greater uncertainty (Lee et al., 2015). Indeed, the literature points to a “bias against novelty” on the grounds that the competitive selection procedures of funding agencies and increasingly risk-averse evaluators prefer safer projects to risky novel research (Boudreau et al., 2012; Wang et al., 2017; Wang et al., 2018). Indeed, diverse teams may be penalized and see their projects rejected as evaluators may find it difficult to assess this diversity (Lamont, 2009), perceiving their projects to be riskier and less feasible (Luukkonen, 2012). In a recent study, Banal-Estañol et al. (2019b) found that structurally diverse teams exhibiting greater heterogeneity in terms of knowledge and skills, education, and/or scientific ability are less likely to receive grants. Evaluators or funding agencies are thus faced with a dilemma in which they have to decide whether or not to allocate scarce funds to structurally diverse teams.

In addition to a team's structural diversity, a further dimension that might prove influential for funding agencies seeking to foster KTT—either explicitly or implicitly—is a team's prior experience of collaboration with the industry. Previous participation in KTT activities generates greater expectations about continuing transfer practices in the future (Bercovitz and Feldman, 2003) as a result of learning by doing or learning by interacting with industry. For instance, D’Este and Patel (2007) found that researchers with prior industry collaborations are more likely to be involved in interactions with industry both in terms of the frequency and variety of interaction channels used. To account for previous collaborations, the authors use a number of joint publications with the industry and the average value of collaborative grants with industry in order to capture the effects of a variety of channels. In addition, the authors suggest that a large number of interactions with industry in the past may indicate the establishment of a personal network of relationships which is likely to have generated enduring mutual trust. Thus, funding agencies that seek to promote knowledge transfer may favor teams with prior experience of industry engagement and those that, therefore, have access to an already established industry network, making them more likely to transfer knowledge in the future. Conversely, if collaborative activities are perceived negatively—because, for instance, evaluators believe that collaborations interfere with the orientation of the proposed research—then past collaborations may be penalized in the grant evaluation process.

### Direct and Indirect Industry Engagements

In this subsection, we differentiate between the *direct* and *indirect* industry engagements of grant applicant teams in the light of team diversity and their prior experience of industry collaborations.

Empirical KTT research to date has focused primarily on formal interaction channels—above all, patents—given their greater data availability and ease of measurement. Yet, there is a growing stream in the literature that concerns itself with less formalized channels (Grimpe and Fier, 2010; Perkmann et al., 2013). Here, we also focus on these less formal channels and explore the relationship established by two types of industry collaboration engaged in by applicant teams with grant evaluation outcomes.

We measure industry engagement by focusing on two types of interactions. First, we examine *direct* engagements (those that manifest during the grant application process), that is, joint or collaborative project applications with industry, whereby an industry/company affiliated member forms a part of the team. If the team members are affiliated to different organizations (i.e., university or industry), the team is likely to exhibit greater diversity in terms of knowledge and skills. As discussed previously, structurally diverse teams are more likely to create novel combinations, which should increase the probability of their producing transferable (or even transferred) output. They can achieve this because of the enhancement of proximity to the market of their research results and the fact that such teams form a part of an established industry network thanks to their ongoing collaboration. Thus, team diversity, proxied here by *direct* engagement, could be a decisive factor in a grant allocation process.

Second, we examine *indirect* engagements (those made prior to the grant application), that is, joint publications with industry. Prior joint publications involving applicant team members and an industry/company affiliated researcher are used, here, as a proxy for the prior collaboration experience (Tijssen et al., 2009). Past collaboration with industry allows teams to enhance their KTT capacity in the future, exploiting mechanisms of learning by doing and an already established network with industry researchers (D’Este and Patel, 2007). Thus, prior experience of collaboration with industry, proxied here by *indirect* engagement, could also be a decider in the allocation of grants. Below, we discuss in further detail our proxies of *direct* and *indirect* industry engagements and how we propose measuring them.

#### Direct Engagement: Collaborative Project Applications With Industry

First, to capture the diversity effect, we consider applications to the funding program in question that include an industry partner. This we refer to as a team's *direct* engagement, being indicative of an ongoing collaborative research project with industry/company at the time of application.

Large-scale empirical studies at the aggregate level find complementarities between public and industry collaboration in R&D projects (David et al., 2000). The synergy between university scientists' research and firms' innovative efforts to create new products and processes is found to be the main driver of U–I collaborations (Mansfield and Lee, 1996).

Applying for competitive research grants in collaboration with firms has benefits as well as costs for both parties. On the one hand, collaboration might attract extra funding from industry (in addition to the grants allocated through public funds) and might provide new perspectives for the collaborating parties (Mansfield, 1995; Lee, 2000). On the other hand, collaboration might have a number of distorting effects, such as the appropriation of research results by industry. Collaboration might also result in delays in the publication of outcomes, which can negatively affect the allocation of public funds to such teams if the funding organization or evaluator perceives dissemination through publications to be a priority. On this particular matter, the literature is divided. Some studies report a link between collaboration and a higher number of publications (Fabrizio and Di Minin, 2008; Azoulay et al., 2009), while others observe a negative impact (Murray and Stern, 2007; Huang and Murray, 2009) or an inverted U-shaped relationship (Crespi et al., 2011; Banal-Estañol et al., 2015).

Banal-Estañol et al. (2013) reported that collaborative research projects with firms result in more scientific output than that produced by non-collaborative projects, provided the partner firm is research intensive. However, it has been argued that collaboration can divert the orientation of a university's research agenda (Florida and Cohen, 1999) and be detrimental to the open science culture (Dasgupta and David, 1994). Similarly, while collaborations with academia may not coincide with the rule of secrecy prioritized by firms, they might have positive effects on the productivity of firms' R&D (Cohen et al., 2002), on their patents, products, and sales (Zucker et al., 2002; Cassiman and Veugelers, 2006) and on their absorptive capacity (Cohen and Levinthal, 1990).

An additional dimension affecting the collaboration decision is the type of research in which researchers engage (i.e., basic vs applied). Drawing on the grant application data of the UK’s Engineering and Physical Sciences Research Council (EPSRC), Banal-Estañol et al. (2019a) found that applied researchers tend to submit more collaborative projects with firms. Furthermore, they found an inverted U-shaped relationship between team size and collaboration with firms and a lower rate of collaboration with teams based in a Russell Group university. Their findings also reveal that collaborative projects with firms are more likely than non-collaborative projects to be funded, yet these collaborative projects have a lower publication rate.

U–I collaborations strengthen diversity and, hence, provide fertile ground for the proliferation of original research. Yet, diversity, while sometimes rewarded, might also be penalized during the grant allocation process as novel research is associated with higher risks. Thus, evaluators may either look favorably on the collaboration and opt to allocate public grants to structurally diverse teams or, equally, they might opt to deny a collaborative project funding due to the aforementioned costs of industry collaborations.

#### Indirect Engagement: Joint Publications With Industry

Joint publications with industry are one of the analytical tools that enable a comparison and assessment of the interactions between universities and industry (Tijssen et al., 2009). Given the confidential nature of contractual engagements, previous studies have used coauthorship as a proxy for collaborative activity (Mindruta, 2013).

Yet, as Katz and Martin (1997) remind us, co-publication data are not without their limitations. For instance, not all joint publications are indicative of an actual U–I collaboration, given that a coauthor might have left the university and affiliated to industry or he or she might have a dual affiliation. Despite these limitations, joint publications are one of the standard means for measuring interorganizational research collaborations, given that the indicator is quantifiable, invariant, and relatively inexpensive to access (Abramo et al., 2009).

An active collaboration may manifest itself in joint U–I publications in peer-reviewed journals and conference proceedings (Melin and Persson, 1996; Tijssen et al., 2009; Tijssen, 2012). Such co-publications include the author's company or industry address, thus allowing the existence and scope of collaborative activity to be identified. Thus, a joint publication provides the information needed for constructing aggregate-level proxy measures for U–I collaboration, both in terms of intensity and magnitude (Tijssen et al., 2009; Tijssen et al., 2016). Abramo et al. (2009) reported a positive influence of joint publications with industry on the research performance of university researchers in Italy. Moreover, a number of studies have found a positive influence of joint publications on the KTT output of universities (D’Este and Patel, 2007; Carayol and Matt, 2004; Wong and Singh, 2013).

Even in such cases where the affiliation of the coauthors may not be immediately visible during the evaluation process (because of either a lack of time or the availability of resources), co-publications may reveal the existence of another type of collaborative activity in the past that applicants explicitly detail in their CVs. Furthermore, forming a part of an industry network as a result of such collaborative activities might be an indicator of greater research visibility which should be readily observable in the review process.

Thus, joint publications with industry/company are useful analytical tools that allow us to proxy for prior industry engagement of a grant applicant team and hence to their ability to transfer knowledge and technology in the future. In the broader research funding framework, we refer to this activity as a team's *indirect* engagement with industry since it is something that has been established prior to the grant application. It is our conjecture that prior experience of collaboration could be decider in a funding process for evaluators that seek to foster U–I relations. Yet, to the best of our knowledge, no study to date has examined the effect of joint publications with industry on the outcomes of the grant evaluation and/or funding allocation process.

In the section that follows, we describe the specific stages making up the grant evaluation and allocation process of the EUROCORES Scheme.

### EUROCORES Grant Evaluation and Allocation Process

European Collaborative Research (EUROCORES) was a broad, complex pan-European grant scheme aimed at supporting collaborative and interdisciplinary research by bringing together national research funding and performing organizations. The program facilitated multinational research collaboration, while funding remained at the national level. EUROCORES was initiated by the ESF in 2001 and included in the European Commission's Sixth Framework Programme (FP6) between 2003 and 2009. Networking and science support activities were later financed by the ESF's member organizations until the EUROCORES scheme was terminated in 2015.

**FIGURE 1:**
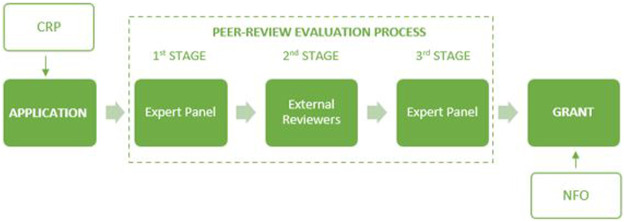
EUROCORES multistage evaluation process. CRP: Collaborative Research Project, NFO: National Funding Organization.

The evaluation of Collaborative Research Project (CRP) proposals in the EUROCORES scheme was a multistage process and included the establishment of an independent, international Expert Panel (see Figure 1). In response to an open call for proposals, outline proposals of about three pages in length were submitted by a team of applicants composed of principal investigators (PIs) and associate partners (APs), coordinated by a project leader (PL) (in principle, a minimum of three PIs from at least three different countries were required).^2^ At this outline proposal stage (First Stage), the Expert Panel was responsible for the sifting of proposals prior to the invitation of full proposals. The panel constituted a group of experts in the relevant scientific domains, brought together for a 1- to 2-day face-to-face meeting to select the proposals via a process of consensus.

At the full proposal stage (Second Stage), each proposal was sent for written external assessment to at least three independent reviewers, including reviewers from outside Europe. Reviewers were asked to fill out an evaluation form comprising 8–10 sections. Each section addressed a different criterion, including the scientific quality of the proposal, the multidisciplinarity of the proposal, the suitability of the PIs and their groups for the project, the complementarity of the groups, the feasibility of the proposal and the budget requested. For each criterion, the reviewers were asked to assign a score on a four- or five-point Likert scale and to add their written comments in the corresponding space. Reviewers were typically given 4 weeks to complete their reports, and the applicants then had the opportunity to reply to the anonymous reviewer reports.^3^

Finally, the written reviewer assessments and the replies submitted by the applicants were considered by the Expert Panel, with scientific quality constituting the main selection criterion. In the last stage of the scientific assessment (Third Stage), the Expert Panel selected the CRPs that were to be recommended to the National Funding Organizations (NFOs) for funding. National eligibility criteria might then have had to be applied to particular applicants from countries that specified “special national requirements” for project applications. Finally, the NFOs were asked to approve funding for the individual teams from their countries in accordance with established national procedures.

## Data and Methodology

### Description of Data

We used research grant data drawn from the EUROCORES funding scheme, provided by the ESF, for the period 2002–2010. The initial data set provides information for 10,533 grant applicants across all scientific domains who teamed up to form a CRP, corresponding to 1,642 outline proposals and 886 full proposals. The process was overseen by a total of 2,246 External Reviewers and 829 panel members. EUROCORES established 47 programs^4^ in a range of disciplines (Life, Earth and Environmental Sciences; Biomedical Sciences; Physical and Engineering Sciences; Humanities and Social Sciences), and about 150M euros was allocated to fund the selected projects.

For each application submitted, the data contained information on the name, year of birth, gender, and institutional affiliation of the applicants and evaluators and reported the date of the application and the amount of funding requested. Information concerning the applicants' *direct* engagement with industry, proxied here by an ongoing project partnership in a CRP with a company-affiliated researcher, can therefore be obtained directly from the EUROCORES database. The data also reported the decisions of and the scores given by the evaluators at the corresponding stage.

Bibliometric data on research publications were extracted from the Web of Science (WoS) Core Collection, a comprehensive, international multidisciplinary bibliographic database. As of 2020, the WoS Core Collection contained over 21,100 international peer-reviewed journals (including open access journals) in 254 WoS journal subject categories, as well as books and conference proceedings. As with its similar counterparts, the WoS database presents a number of drawbacks, including the overrepresentation of English-language journals to the detriment of other languages and the underrepresentation of publications in the Social Sciences and Arts and Humanities (Mongeon and Paul-Hus, 2016; Vera-Baceta et al., 2019). Nevertheless, the WoS database continues to be one of the main sources for bibliometric analyses.

The bibliographic record of each publication contains, among other details, the authors' institutional affiliation, which enables us to identify joint publications with industry (or company) affiliated researchers. To identify coauthors affiliated to a company, we ran a computer code that was able to detect abbreviations or extensions related to a private entity, such as, “ltd., Inc., GmbH, SRL, public limited company, limited liability company”. Through this coauthorship relation, we were able to detect the applicants' *indirect* engagement with industry prior to the grant application. In our database, 48,458 out of a total of 707,158 applicant publications include at least one coauthor with a private company affiliation (i.e., 6.8%).

### Econometric Treatment

To capture both the *diversity* and *experience* effects, we examined the influence of the *direct* (project partnership) and *indirect* (coauthorship) industry engagement of the applicant teams on the outcomes of the three consecutive stages in the grant peer-review process: that is, the first Panel Stage (First Stage), the External Reviewer Stage (Second Stage), and the second Panel Stage (Third Stage). In our analyses, we do not consider the final decision of the NFOs to either fund or not fund the project, given that this ultimate stage is only marginally different from the Third Stage of selection. In the majority of cases, the NFOs adhered to the funding recommendations of the Expert Panels.^5^

In the First Stage, we estimate the probability of passing to the second stage, in which applicants were invited to submit the full proposal. As the dependent variable in this stage is binary (i.e. passed or not), we use a probit model. In the Second Stage, we used an OLS model since the dependent variable is defined as the average review score awarded by the External Reviewers. In the analysis reported here, we consider the average reviewer score calculated over the following five questions^6^: Q1—scientific quality of the proposed research, Q2—multidisciplinarity of the proposal, Q3—suitability of the PIs for the project, Q4—the level of collaborative interaction between the groups in the project, and Q5—feasibility of the project and appropriateness of methodology. We had to standardize the review scores because in some of the years, scores were awarded on a 4-point Likert scale, while in others, these were awarded on a five-point scale. Finally, in the Third Stage, regarding recommendation for funding, we again used a probit estimate since the dependent variable is binary (i.e., passed or not).

We corrected for any potential sample selection bias by following Heckman's two-stage estimation procedure (Heckman, 1979), using the inverse Mills ratio (IMR)^7^. The IMR generated from the first probit estimation is included in the OLS estimation as an additional explanatory variable. Regressions were estimated with heteroscedasticity-consistent standard errors clustered at the program-level and include year, scientific field, and program fixed effects.

### Variable Specification

#### Main Explanatory Variables

Our main explanatory variables concern the *direct* and *indirect* industry engagements of the applicant teams, which shed light on the diversity and experience effects. Direct involvement is proxied by the existence of team members in a CRP with a company affiliation during the EUROCORES grant application. Thus, *private partner* is a binary variable taking a value of 1 if the applicant team has at least one team member with a company affiliation and 0 if otherwise. Indirect involvement is proxied by evidence of a team's joint publication with industry prior to the EUROCORES grant application. Thus, *joint publication* is a binary variable taking a value of 1 if the applicant team has at least one member with a publication coauthored with a company-affiliated researcher and 0 if otherwise.

#### Control Variables

We included several control variables measured at the applicant team-level and evaluator-level and which may affect the outcome of the evaluation process (see Table 1 for variable descriptions). Bibliometric indicators are constructed through the individual data retrieved from the WoS database. We measured *Productivity* by the number of total publications of the team members normalized by the team size (in logs). *Star scientist* indicates whether the team includes at least one of the most cited researchers, corresponding to the top 1% of a specific discipline and the year of citation distribution. To measure *Research diversity*, we used the *Blau index,* given by 1 − ∑s_i_^2^, where s_i_ is the share of publications in a WoS subject category i, calculated with respect to other team members. We clustered 254 WoS subject categories into 21 categories in line with the literature (Glänzel and Schubert, 2003; Leydesdorff and Rafols, 2009). We also accounted for different proximity measures to the evaluator. *Cognitive proximity* is the share of overlapping clustered WoS subject categories between team members and the evaluator (either the Expert Panel members or the External Reviewers depending on the stage of evaluation). *Network proximity* is a dummy variable taking a value of 1 if the team and the panel members share at least one coauthor in common prior to the grant application^8^.

**TABLE 1:** Variable descriptions.

Variable	Description
Dependent variables	
Passed First Stage	Dummy equal to 1 if the project passed the first stage of the selection process (from outline to full proposal stage)
Reviewer score	Average reviewer score to questions Q1–Q5 (z-scores)
Passed Third Stage	Dummy equal to 1 if the project passed the third stage of the selection process and is thus recommended for funding to NFOs
Applicant Team Characteristics	
Joint publication	Dummy equal to 1 if the team includes at least one member having a co-publication with a company-affiliated researcher prior to the grant application
Private partner	Dummy equal to 1 if the team includes at least one member having a private sector affiliation
Productivity	Average number of publications of the team members (in logs)
Star scientist	Dummy equal to 1 if the team includes at least one of the most cited applicants based on the discipline–year citation distribution (top 1%)
Cognitive proximity	Share of overlapping WoS subject categories between team members and Panel Members/Reviewers (254 WoS subject categories clustered into 21)
Research diversity	Average Blau index of team members (1 − ∑ s_i_^2^) where s_i_ is the share of publications in clustered WoS subject category i)
Network proximity	Dummy equal to 1 if the team and panel members have at least one coauthor in common
Female ratio	Share of female members within a team
Age	Average age of team members (in logs)
Shanghai ranking	Dummy equal to 1 if the team includes at least one member affiliated to a university in the Shanghai Ranking top 100 (base year 2005)
Past EUROCORES grant	Dummy equal to 1 if the team includes at least one member awarded a EUROCORES project in previous years
Team size	Number of researchers within a team composed of a Project Leader, Principal Investigators, and Associate Partners (in logs)
Number of countries	Number of distinct participating countries within a team (in logs)
Budget requested	Total budget requested for the project normalized by team size (Euro, in logs)
Expert Panel Characteristics	
Joint publication panel	Dummy equal to 1 if the Expert Panel includes at least one member having a co-publication with a company-affiliated researcher
Female ratio panel	Share of female members within a Panel
Productivity panel	Average number of publications of Panel Members (in logs)
Age panel	Average age of Panel Members (in logs)
Shanghai ranking panel	Dummy equal to 1 if the Panel includes at least one member affiliated to a university in the Shanghai Ranking top 100 (base year 2005)
Panel size	Number of members within a Panel (in logs)
Panel workload	Number of assessed projects normalized by panel size (in logs)
External Reviewer Characteristics	
Joint publication reviewer	Dummy equal to 1 if the External Reviewer has a co-publication with a company-affiliated researcher
Female reviewer	Dummy equal to 1 if the External Reviewer is female
Productivity reviewer	Number of publications of the External Reviewer (in logs)
Age reviewer	Age of the External Reviewer (in logs)
Shanghai ranking reviewer	Dummy equal to 1 if the External Reviewer is affiliated to a university in the Shanghai Ranking top 100 (base year 2005)

In addition, we include the following demographic characteristics of the team: *Female ratio* controls for the gender composition of a team calculated as the share of females in the applicant team. *Age* is the average age of team members (in logs). *Shanghai ranking* is a binary variable that accounts for institutional prestige and takes a value of 1 if the team includes at least one member affiliated to a university included in the Shanghai Ranking top 100 (base year taken as 2005).

Moreover, we consider a dummy called *Past EUROCORES grant* which takes a value of 1 if the team includes at least one member who has obtained a EUROCORES grant in the past. This variable accounts for a phenomenon similar to the so-called Matthew Effect (Merton, 1968) and also for the ‘learning by doing’ effect. *Team size* is the number of PIs within the applicant team (in logs) who lead an individual project within a CRP^9^. We control also for *Number of countries*, which refers to the number of distinct participant countries within a team (in logs), since country diversity is one of the prerequisites of EUROCORES. Finally, concerning applicant team characteristics, we consider the *Budget requested* normalized by team size (in logs). For the Third Stage, we also include *Reviewer scores mean as* an explanatory variable since the final Expert Panel decision is contingent on the scores given by the reviewers. To calculate this, standardized average review scores were divided by the number of reviewers corresponding to the CRP. To account for the divergence of scores given by different reviewers to the same CRP, we include the variable *Reviewer scores variance*.

A number of studies show that the grant decision process is not solely influenced by applicant characteristics but also by the characteristics of the evaluators (Mutz et al., 2012; van Arensbergen et al., 2014; Gallo et al., 2016). For the First and Third Stages of evaluation, we therefore take into consideration Expert Panel characteristics. Given that the panel comprises a group of scientists, the measure has to be aggregated. First, we control for the panel's *indirect* engagement by considering the variable *Joint publication panel*.^10^ Then, we control for the panel's gender by means of the *Female ratio panel*. Next, we introduced the variables of *Productivity panel, Age panel*, and *Shanghai ranking panel*. All these controls are calculated in the same way as for the applicant teams. In addition, we consider *Panel size*, that is, the number of members sitting on a panel (in logs). Finally, we take into account *Panel workload*, which is the number of assessed projects normalized by *Panel size* (in logs).

For the Second Stage of evaluation, we consider External Reviewer characteristics, measured in this instance at the individual level. Following the same procedure, we account for gender by means of the variable *Female reviewer*, and include variables considering *Productivity reviewer*, *Age reviewer*, and *Shanghai ranking reviewer.*

Finally, we include scientific discipline, year, and program fixed effects using dummies. In line with the ESF's classification, we cluster CRPs into three scientific disciplines, namely “physics and engineering”, “life and biomedical sciences,” and “social sciences and humanities,” to obtain a more even distribution of the disciplines. We control for the project application year as well as for the EURCORES program representing the relevant CRPs.

### Descriptive Statistics

Tables 2, 3, 4, 5, 6 show the descriptive statistics for the applicant teams and evaluators. In this subsection, we present the descriptive statistics for some of the variables outlined above. The percentage of project applications that passed the First Stage of selection (from outline proposal to full proposal) is about 39%. At the beginning of this stage, 53% of teams present at least one team member with a joint publication with a company. Around 4% of the teams have at least one team member with a company affiliation. On average, the number of publications were 11 and 6.5% of teams contain a star scientist. As for the teams' demographics, on average, the share of females in the applicant teams was 19.5% and the average age of applicants was 47 years. The average number of members in a team was 6.8, and the budget requested per capita was 271,765 euros. Of all the outline proposals, about 48% are in life and biomedical sciences, 26% in physics and engineering, and 26% in social sciences and humanities.

**TABLE 2:** Descriptive statistics of applicants—First stage.

Statistic	Observations	Mean	Std dev	Min	Max
Passed First Stage	1,378	0.388	0.487	0	1
Joint publication	1,378	0.533	0.499	0	1
Private partner	1,378	0.038	0.191	0	1
Productivity	1,378	11.033	19.604	0	186
Star scientist	1,378	0.065	0.246	0	1
Cognitive proximity	1,378	0.368	0.348	0	1
Research diversity	1,378	0.440	0.317	0	1
Network proximity	1,378	0.025	0.155	0	1
Female Ratio	1,378	0.195	0.208	0	1
Age	1,378	46.746	4.585	34	66,333
Shanghai Ranking	1,378	0.577	0.494	0	1
Past EUROCORES grant	1,378	0.134	0.341	0	1
Team size	1,378	6.800	4.572	3	51
Number of countries	1,378	4.389	1.894	2	16
Budget requested	1,378	271,765.000	211,800.400	10,417	4,448,720
Life and biomedical sciences	1,378	0.478	0.500	0	1
Physics and engineering	1,378	0.260	0.439	0	1
Social sciences and humanities	1,378	0.262	0.440	0	1

**TABLE 3:** Descriptive statistics of applicants—Second Stage.

Statistic	Observations	Mean	Std dev	Min	Max
Average reviewer score	1,930	0.003	0.793	−2.704	1.066
Joint publication	1,938	0.602	0.490	0	1
Private partner	1,938	0.024	0.154	0	1
Productivity	1,938	12.467	19.470	0	145
Star scientist	1,938	0.084	0.278	0	1
Cognitive proximity	1,938	0.331	0.434	0	1
Research diversity	1,938	0.480	0.308	0	1
Female ratio	1,938	0.166	0.188	0	1
Age	1,938	46.897	4.596	34	66.333
Shanghai ranking	1,938	0.685	0.465	0	1
Past EUROCORES grant	1,938	0.214	0.410	0	1
Team size	1,938	7.126	4.329	3	51
Number of countries	1,938	4.678	1.846	2	15
Budget requested	1,938	276,555.400	173,829.500	10,417	2,557,539
Life and biomedical sciences	1,938	0.377	0.485	0	1
Physics and engineering	1,938	0.313	0.464	0	1
Social sciences and humanities	1,938	0.310	0.463	0	1

**TABLE 4:** Descriptive statistics of applicants—Third Stage.

Statistic	Observations	Mean	Std dev	Min	Max
Passed Third Stage	533	0.612	0.488	0	1
Joint publication	533	0.623	0.485	0	1
Private partner	533	0.024	0.154	0	1
Productivity	533	13.193	20.092	0	145
Star scientist	533	0.081	0.273	0	1
Cognitive proximity	533	0.445	0.360	0	1
Research diversity	533	0.487	0.305	0	1
Network proximity	533	0.032	0.176	0	1
Female ratio	533	0.170	0.193	0	1
Age	533	46.943	4.607	34	66.333
Shanghai ranking	533	0.685	0.465	0	1
Past EUROCORES grant	533	0.205	0.404	0	1
Team size	533	7.024	4.237	3	51
Number of countries	533	4.689	1.869	2	15
Budget requested	533	276,625.500	176,882.300	10,417	2,557,539
Reviewers scores mean	525	−0.010	0.547	−2.233	0.998
Reviewers scores variance	524	0.498	0.535	0	3.700
Life and biomedical sciences	533	0.375	0.485	0	1
Physics and engineering	533	0.319	0.467	0	1
Social sciences and humanities	533	0.306	0.461	0	1

**TABLE 5:** Descriptive Statistics of Expert Panel [41 panel groups].

Statistic	Observations	Mean	Std dev	Min	Max
Joint publication panel	41	0.110	0.169	0	1
Female ratio panel	41	0.184	0.126	0	0.455
Productivity panel	41	11.011	26.871	0	166.333
Age panel	41	51.024	3.383	43.231	58.200
Shanghai ranking panel	41	0.927	0.264	0	1
Panel size	41	12.293	4.360	4	30
Panel workload	41	2.921	2.649	0.385	14.286

**TABLE 6:** Descriptive statistics of external reviewers.

Statistic	Observations	Mean	Std dev	Min	Max
Joint publication reviewer	1,938	0.138	0.345	0	1
Female reviewer	1,938	0.166	0.372	0	1
Productivity reviewer	1,938	6.569	15.995	0	177
Age reviewer	1,938	48.496	10.181	30	85
Shanghai ranking reviewer	1,938	0.269	0.443	0	1
Average reviewer score	1,930	0.003	0.793	−2.704	1.066

At the beginning of the Second Stage, following the initial sifting by the Expert Panel, teams with *indirect* engagement increased to 60%, and the share of teams with *direct* engagement dropped to 2.4%. The average productivity increased to 12.4 publications per applicant. At this stage, we observed a decline in the share of females to 16.6%.

At the end of the Third Stage of selection, 61% of full proposals were recommended for funding to the NFOs. Teams with *indirect* engagement increased to 62%, and the share of teams with *direct* engagement remained at 2.4%. The average productivity increased to 13 publications.

As for the reviewers, the share with joint publications was about 14%, while Expert Panels including at least one member with a joint publication with a company stood at about 11%. On average, the Expert Panels contained 12 members, and the number of projects evaluated per panel member was about three.

The correlation matrix presented in Table 7 shows the correlation coefficients between the variables concerning the applicant team at the First Stage.

**TABLE 7:** Pairwise correlation matrix.

		1	2	3	4	5	6	7	8	9	10	11	12	13
1	Joint publication													
2	Private partner	0.02												
3	Female ratio team	−0.04	−0.06*											
4	Productivity team	0.38**	0.01	−0.04										
5	Star scientist team	0.21**	0.03	−0.03	0.44**									
6	Cognitive proximity team	0.32**	−0.03	−0.02	0.32**	0.08**								
7	Research diversity team	0.39**	−0.03	0.00	0.22**	0.05	0.60**							
8	Network proximity	0.14**	−0.03	−0.02	0.15**	−0.00	0.11**	0.10**						
9	Team size	0.22**	0.08**	−0.01	0.09**	0.17**	0.17**	0.23**	0.11**					
10	Age team	0.10**	−0.03	−0.09**	0.08**	−0.00	0.06*	0.06*	0.04	−0.06*				
11	Shanghai ranking team	0.10**	0.01	−0.04	0.03	0.05	0.11**	0.12**	0.05	0.15**	0.04			
12	Past EUROCORES grant team	0.12**	−0.04	−0.11**	0.11**	0.04	0.12**	0.19**	0.07**	0.09**	0.03	0.06*		
13	Budget requested	0.03	−0.01	0.04	0.05*	−0.03	−0.02	0.04	−0.01	−0.16**	0.10**	0.03	0.03	
14	Number of countries	0.25**	0.02	−0.01	0.11**	0.10**	0.22**	0.28**	0.14**	0.69**	0.07**	0.22**	0.14**	−0.07**

Pairwise correlation matrix of applicant team characteristics for the First Stage of evaluation; * indicates *p* < 0.05; ** indicates *p* < 0.01.

## Econometric Results

We investigated the impacts of team diversity and prior collaboration experience of EUROCORES applicants on the decisions of the evaluators at consecutive stages of the evaluation process. The results of the estimations are reported in Tables 8, 9, 10. We used different model specifications by introducing variables related to the applicant team and the evaluators stepwise. All regressions were estimated with heteroscedasticity-consistent standard errors clustered at the program-level and included year, scientific field and program fixed effects. As a cautionary note, the following results allowed us to observe the correlation between the dependent and independent variables; they cannot be confidently interpreted as causal.

**TABLE 8:** First Stage—Probit regression results.

Dependent variable: Passed First Stage			
	(1)	(2)	(3)
Joint publication	0.086***	0.082***	0.050*
	(0.026)	(0.025)	(0.030)
Private partner	−0.060	−0.077	−0.092
	(0.079)	(0.078)	(0.071)
Productivity	0.035	0.021	0.021
	(0.033)	(0.030)	(0.032)
Star scientist	0.059	0.064	0.051
	(0.057)	(0.061)	(0.056)
Cognitive proximity	0.123***	0.126***	0.116**
	(0.047)	(0.047)	(0.049)
Research diversity	−0.036	−0.061	−0.139***
	(0.052)	(0.053)	(0.043)
Network proximity	−0.032	−0.046	−0.185*
	(0.103)	(0.106)	(0.096)
Female ratio		−0.187**	−0.197***
		(0.074)	(0.071)
Age		−0.162	−0.211
		(0.167)	(0.172)
Shanghai ranking		0.126***	0.096***
		(0.031)	(0.032)
Past EUROCORES grant			0.082*
			(0.050)
Team size			0.120***
			(0.045)
Number of countries			0.138***
			(0.050)
Budget requested			0.030
			(0.020)
Joint publication panel			−0.337
			(0.353)
Female ratio panel			−0.103
			(0.215)
Productivity panel			0.113
			(0.105)
Age panel			0.438
			(0.418)
Shanghai ranking panel			−0.005
			(0.177)
Panel size			−0.125**
			(0.061)
Panel workload			−0.216***
			(0.048)
Program fixed effects	yes	yes	no
Year dummies	yes	yes	yes
Domain dummies	yes	yes	yes
AIC	1,574.516	1,552.338	1,547.474
BIC	1830.707	1824.215	1714.782
Log likelihood	−738.258	−724.169	−741.737
Deviance	1,476.516	1,448.338	1,483.474
Num. obs	1,378	1,378	1,378

Robust standard errors in parentheses, clustered at program-level. ***, **, * indicate significance at the 1, 5, and 10% levels, respectively.

**TABLE 9:** Second Stage—OLS regression results.

Dependent variable: Reviewer score						
	(1)	(2)	(3)	(4)	(5)	(6)
Joint publication	−0*.*024	−0*.*099^∗∗^	−0*.*031	−0*.*079	−0*.*047	−0*.*070
	(0*.*045)	(0*.*048)	(0*.*045)	(0*.*049)	(0*.*047)	(0*.*052)
Private partner	−0*.*062	−0*.*058	−0*.*061	−0*.*058	−0*.*095	−0*.*062
	(0*.*123)	(0*.*122)	(0*.*122)	(0*.*122)	(0*.*124)	(0*.*128)
Productivity	0*.*169^∗∗∗^	0*.*152^∗∗∗^	0*.*149^∗∗∗^	0*.*141^∗∗∗^	0*.*160^∗∗∗^	0*.*145^∗∗∗^
	(0*.*038)	(0*.*038)	(0*.*039)	(0*.*039)	(0*.*040)	(0*.*042)
Star scientist	−0*.*159^∗∗^	−0*.*201^∗∗∗^	−0*.*154^∗∗^	−0*.*180^∗∗∗^	−0*.*164^∗∗^	−0*.*178^∗∗^
	(0*.*068)	(0*.*068)	(0*.*068)	(0*.*068)	(0*.*068)	(0*.*069)
Cognitive proximity	−0*.*148^∗∗∗^	−0*.*168^∗∗∗^	−0*.*151^∗∗∗^	−0*.*162^∗∗∗^	−0*.*125^∗∗^	−0*.*129^∗∗^
	(0*.*051)	(0*.*051)	(0*.*051)	(0*.*051)	(0*.*053)	(0*.*053)
Research diversity	0*.*047	0*.*018	0*.*032	0*.*024	−0*.*019	0*.*009
	(0*.*072)	(0*.*072)	(0*.*072)	(0*.*072)	(0*.*075)	(0*.*080)
Female ratio			−0*.*351^∗∗∗^	−0*.*242^∗∗^	−0*.*328^∗∗∗^	−0*.*247^∗^
			(0*.*104)	(0*.*112)	(0*.*105)	(0*.*130)
Age			0*.*108	0*.*188	0*.*062	0*.*142
			(0*.*191)	(0*.*193)	(0*.*193)	(0*.*207)
Shanghai ranking			0*.*140^∗∗∗^	0*.*076	0*.*125^∗∗∗^	0*.*084
			(0*.*040)	(0*.*047)	(0*.*041)	(0*.*056)
Past EUROCORES grant					0*.*087	0*.*040
					(0*.*057)	(0*.*072)
Team size					0*.*014	−0*.*023
					(0*.*065)	(0*.*074)
Number of countries					0*.*080	0*.*025
					(0*.*082)	(0*.*097)
Budget requested					0*.*004	−0*.*009
					(0*.*031)	(0*.*034)
Joint publication reviewer					−0*.*018	−0*.*017
					(0*.*061)	(0*.*061)
Female reviewers					−0*.*044	−0*.*040
					(0*.*048)	(0*.*048)
Productivity reviewer					−0*.*050^∗∗^	−0*.*050^∗∗^
					(0*.*023)	(0*.*023)
Age reviewer					0*.*087	0*.*086
					(0*.*085)	(0*.*085)
Shanghai ranking reviewer					0*.*016	0*.*015
					(0*.*039)	(0*.*039)
Inverse Mills Ratio		−0*.*449^∗∗∗^		−0*.*299^∗∗^		−0*.*252
		(0*.*093)		(0*.*117)		(0*.*238)
Program fixed effects	yes	yes	yes	yes	yes	yes
Year dummies	yes	yes	yes	yes	yes	yes
Domain dummies	yes	yes	yes	yes	yes	yes
Observations	1,930	1,930	1,930	1,930	1,930	1,930
R2	0.143	0.154	0.155	0.157	0.161	0.162
Adjusted R2	0.123	0.133	0.133	0.136	0.136	0.136
Residual Std. Error	0.742	0.738	0.738	0.737	0.737	0.737
F Statistic	7.005***	7.435***	7.163***	7.170***	6.308***	6.219***

Robust standard errors in parentheses, clustered at program-level. ***, **, * indicate significance at the 1, 5, and 10% levels, respectively.

**TABLE 10:** Third Stage—Probit regression results.

Dependent variable: Passed Third Stage				
		(1)	(2)	(3)
Joint publication		−0.050	−0.054	−0.015
		(0.057)	(0.056)	(0.064)
Private partner		−0.354***	−0.378***	−0.165
		(0.123)	(0.120)	(0.234)
Productivity		0.102**	0.098**	−0.047
		(0.048)	(0.047)	(0.040)
Star scientist		−0.011	−0.007	0.164**
		(0.090)	(0.083)	(0.083)
Cognitive proximity		0.023	0.029	−0.043
		(0.090)	(0.089)	(0.115)
Research diversity		0.092	0.071	0.246*
		(0.103)	(0.096)	(0.146)
Network proximity		0.209***	0.202***	0.323***
		(0.055)	(0.060)	(0.052)
Female ratio			−0.089	0.040
			(0.144)	(0.161)
Age			−0.186	−0.220
			(0.315)	(0.390)
Shanghai ranking			0.074	−0.073
			(0.072)	(0.101)
Past EUROCORES grant				0.024
				(0.068)
Team size				−0.087
				(0.083)
Number of countries				0.090
				(0.112)
Budget requested				0.094**
				(0.045)
Reviewers scores mean				0.887***
				(0.136)
Reviewers scores variance				0.074
				(0.067)
Joint publication panel				0.531
				(0.549)
Female ratio panel				−0.126
				(0.301)
Productivity panel				−0.153
				(0.156)
Age panel				−1.142**
				(0.559)
Shanghai ranking panel				0.024
				(0.080)
Panel size				−0.405***
				(0.084)
Panel workload				−0.257***
				(0.077)
Program fixed effects	yes	yes		no
Year dummies	yes	yes		yes
Domain dummies	yes	yes		yes
AIC	696.119	698.808		504.598
BIC	897.210	912.734		649.489
Log likelihood	−301.060	−299.404		−218.299
Deviance	602.119	598.808		436.598
Num. obs	533	533		524

Robust standard errors in parentheses, clustered at the program level. ***, **, * indicate significance at the 1, 5; and 10% levels, respectively.

### First Stage

Table 8 reports the results of the probit regression corresponding to the First Stage of selection by the Expert Panel. Coefficients are the marginal effects calculated by setting all variables at their mean values.

In the case of our main variables of interest, the variable capturing the teams' prior experience of collaboration is significant at the First Stage, whereas the variable accounting for the teams' structural diversity is not significant. The *Joint publication* coefficient (indirect engagement) is significantly positive across all models. More specifically, the coefficient of *Joint publication* in Model 3 is 0.05 (*p* < 0.1), implying that the presence in a team of at least one member with a joint publication with a company-affiliated researcher increases the probability of passing the first selection stage by 0.05 compared to a team with no members presenting a joint publication with a company-affiliated researcher. In other words, teams incorporating at least one member with a joint publication with a company are more likely to pass from the outline to the full proposal stage, supporting the argument that teams having prior collaboration experience with industry are more likely to be “rewarded.” On the other hand, we do not observe a significant effect of the *Private partner* variable (direct engagement), which seems to indicate that the diversity argument is not supported.

Overall, the variables reflecting applicants' scientific productivity (i.e. the total number of publications and star scientists) are not significant. At this initial stage, the panel characteristics and the more readily observable characteristics of the applicants, such as research diversity, proximity to the panel, and being affiliated to a top-academic institution, matter the most.

An examination of the bibliometric indicators in the full model specification shows that the coefficient measuring the applicant team's *Cognitive proximity* to a panel member is significantly positive, whereas the coefficients of *Network proximity* and *Research diversity* are significantly negative. However, the team *Productivity* coefficient is nonsignificant at this stage.

The coefficient measuring the share of females is significantly negative, indicating that higher shares of females within teams reduce the likelihood of passing the First Stage. This finding is in line with the literature concerning gender disparities in the grant peer review process (van der Lee and Ellemers, 2015; Shannon et al., 2019; Witteman et al., 2019).^11^ On the other hand, having at least one team member affiliated to a prestigious university, having obtained a EUROCORES grant in the past, and the team size and number of countries represented in the team increase the likelihood of success at this stage, as their coefficients are significantly positive.

Finally, in the case of the panel-level characteristics, we find a negative impact of *Panel size* and *Panel workload*, the latter reflecting the level of competition within a given program, that is, the higher the number of projects per panel, the lower the probability of passing this stage.

### Second Stage

Table 9 reports the results of the OLS regression corresponding to the Second Stage of evaluation by the External Reviewers. As explained above, we control for potential sample selection bias by using a two-step Heckman procedure in some model specifications (i.e., Models 2, 4, and 6). Consequently, the results are reported without and with IMRs.

At this stage, External Reviewers are insensitive to variables reflecting industry engagement both in terms of a team's prior collaboration experience and the involvement of an industry partner in a team. When we correct for potential selection bias in the full model specification, we do not observe a significant relationship between the variables *Joint publication* or *Private partner* with the scores awarded by the reviewers.

Moreover, unlike the First Stage, at this stage of the evaluation, the variables reflecting scientific productivity (i.e., team productivity, star scientist, and reviewer productivity) have a significant relation with the reviewers' scores. Across all models, the variable *Productivity* presents a positive significant coefficient suggesting that a higher number of publications is associated with higher reviewer scores. On the other hand, having a *Star scientist* in the team, *Cognitive proximity* to the reviewer, and a higher *Female ratio* lower the likelihood of obtaining higher review scores as their coefficients are significantly negative. Here, our finding regarding cognitive distance is in line with that of Gallo et al. (2016), suggesting that a closer intellectual distance between the reviewer and the applicant leads to less favorable evaluations.

Finally, we find a negative relation between a reviewer's productivity and the scores awarded to the proposals, that is, the higher a reviewer's productivity, the lower the score given.

### Third Stage

Table 10 reports the results of the probit regression corresponding to the Third Stage of selection by Expert Panel members. Coefficients are the marginal effects calculated by setting all variables at their mean values.

The variables concerning industry engagement (i.e., *Joint publication* and *Private partner*) are not significant. The negative impact of industry engagement as it relates to diversity is only observed in limited specifications. In the full model specification, having a private partner in a team does not significantly affect success in the Third Stage of selection (i.e., obtaining a funding recommendation).

Overall, in this last stage of the process, our findings are in line with the “old boy” network effect (Rose, 1989; Travis and Collins, 1991), since the network proximity of teams to the evaluators is highly determinant of being selected. Likewise, the review outcome of the previous stage is also an important determinant of passing the last stage of selection.

At this stage, a team's *Productivity* is no longer significant while the variable *Star scientist* becomes positively significant. In the Third Stage, the *Reviewer scores mean* has, as expected, a significant positive influence on the outcome, given that the Expert Panel adheres primarily to the reviewer scores awarded in the previous stage. *Network proximity* also has a significant positive impact suggesting that having a coauthor in common with a panel member increases the likelihood of acceptance in this last stage of selection. We also observe a positive impact of the *Budget requested*.

Finally, when we consider panel-level characteristics, we find significant negative effects of *Age panel*, *Panel size*, and *Panel workload*.

## Discussion and Conclusion

This paper has examined the determinants of a multistage grant allocation system as implemented by different sets of evaluators (i.e., Expert Panel and External Reviewers) active at different points in the process. Specifically, we analyzed the impacts of the applicant teams' *direct* industry engagement (diversity effect) and *indirect* industry engagement (experience effect) on the outcomes at each stage in the evaluation.

Our results indicate that scientific productivity is not always the main selection criteria in the grant allocation process. This is in line with previous studies suggesting that the funding outcome may be influenced by the individual characteristics of the applicants (unrelated to their scientific productivity) (Wennerås and Wold, 1997; Grimpe, 2012) or by the attributes of the project evaluators (Gallo et al., 2016). Given that the knowledge transfer goals of STI policy can be achieved, in part, by means of competitive grants, it is our conjecture that the KTT-related attributes of applicant teams might also affect the grant allocation process. Here, we have argued that this can be operationalized by considering two indicators related to industry engagement: 1) whether the applicant team exhibits diversity (*direct* engagement), proxied by the presence of an industry/company-affiliated member in the collaborative research project—in this respect, structurally diverse teams in terms of their knowledge and skills are more likely to produce novel combinations with a high potential for transfer due to direct proximity with the industry network; and 2) whether the team has past experience of collaboration (*indirect* engagement), proxied by the presence of a member in the applicant team with joint publications with industry prior to the grant application. We argue that prior collaboration with industry enhances the potential to transfer in the future, something that can be guaranteed or facilitated, for instance, by means of the informal networks established with industry researchers during the collaborative relation or by means of learning by doing.

Although we found some evidence of *indirect* engagement to support the experience effect, we are unable to identify any significant impact of *direct engagement* to support the diversity effect on the grant allocation process. The reason why prior experience might be more valued than having an industry partner in an applicant team appears to be that it does not constitute a direct threat of the appropriation of research results by industry. Moreover, occupying a position in an established industry network thanks to prior collaborations (proxied here by co-publications) might be perceived by evaluators as a more reliable indicator of future knowledge transfer activities. Such network visibility can be readily captured by the Expert Panel when screening the applicants' CVs in the first stage of selection, at a point in the process when the project content remains somewhat limited because only outline, and not full, proposals are being evaluated. This means that panel members might, at this stage, be basing their selections on the more visible attributes of an applicant team. Yet, despite threats of industry appropriation, direct engagement may also be valued on the grounds that diverse teams are more likely to produce novel research (Arts and Veugelers, 2015). The trade-off between novelty and risk of appropriation of transferable research outcomes might go some way to explain why we do not observe any significant effect of *direct* engagement.

The study reported here contributes to the literature by providing an examination of the impact of industry engagement on the grant allocation process with a particular focus on the effects of an applicant team's *diversity* and *experience*. To the best of our knowledge, the only study to date that has attempted to examine the effects of industry collaboration is Banal-Estañol et al.'s (2019a), and here in the present study, only in part, as it does not constitute the primary focus of the authors' analysis. Our study, therefore, builds on and extends this earlier investigation by incorporating the dimension of *indirect* engagement in an effort to account for collaborations established in the past.

Another important contribution made by the present study is the fact that our original and particularly rich database has allowed us to observe a multistage, multidisciplinary evaluation process at a more granular level within a pan-European grant scheme. Previous studies have tended to concentrate their research on a single country and a single discipline and have restricted themselves to a single stage in an evaluation process. However, grant allocation is rarely a single-step process in which funding organizations decide whether or not to award public research funds; rather, different actors are engaged in the process, and their decisions are likely to exhibit a range of heterogeneities, as shown in our study. In terms of the methodology employed, this study contributes to the wider literature by developing an aggregated approach and conducting its analysis at the team level, whereas most previous studies of grant allocation consider individual-level data. It should be noted, however, that Banal-Estañol et al. (2019b) is an exception in this respect as their study also focuses on the team dimension.

Despite the conjectures we draw above, our empirical analyses are unable to provide a definitive answer to understand why *direct* and *indirect* engagements lead to different outcomes at different stages of the grant application process. Further qualitative investigation is needed to understand what goes in this black box. Additional conjectures might be made to explain stage-specific heterogeneities, including such factors as the differences in group vs individual decision-making processes (Olbrecht and Bornmann, 2010), and the impact of limitations of the information and time made available to evaluators when having to examine applicant profiles and project proposals. Here, the differences between the evaluators engaged at distinct stages of the process in terms of their characteristics, objectives, constraints, and access to relevant information have meant that different evaluation mechanisms have been operational in consecutive stages. The Panel Stage is representative of a group decision-making process under the constraints of time, whereas the External Reviewers operated alone when assessing the proposals and were given more time to evaluate projects and applicants. Additionally, at the first stage of application, panel members evaluate only the outline proposal, and thus, they do not have access to the information that will be included in the full proposal. This contrasts with the information available to the reviewers, which is considerably more detailed with regard to both the project content and the applicants. This explains why at the early stages of selection, more visible quality signals, including university affiliation (Shanghai Ranking) and previous grants held, are significant determinants of selection, whereas in later stages, when the full proposal contents are available, specific quality measures, such as the number of publications and reviewer scores, acquire greater weightage.

A further limitation of our study relates to the different mechanisms that might impact industry collaboration. Here, the set of mechanisms affecting the evaluators' perceptions of the *direct* and *indirect* collaborations of applicant teams could be much broader. To measure *direct* engagement, we used ongoing collaborations with an industry partner during the grant application and related this attribute to a team's structural diversity. Yet, it is evident that in doing so, we employ a rather narrow approach to diversity, given that structural diversity might be measured by using other team attributes as the diversity literature illustrates. Moreover, *direct* industry involvement might capture aspects other than diversity that is related to the scope of a proposed project, i.e., whether the project is more application oriented or whether it seeks to develop solutions for industrial use. Likewise, to account for *indirect* engagement, and hence for the experience effect, different proxies of prior industry engagement, such as patents and know-how licenses, consultancy agreements, spin-off formations, and researcher mobility, can be used in addition to co-publications.

Our research has a number of implications for policy makers and research funding agencies. In a knowledge-based economy, STI policies aim to foster KTT by employing different instruments, including competitive grants. Although, the development of U–I collaboration was not one of the explicit aims of the EUROCORES funding scheme, the transfer of research outcomes was a dimension that was sometimes referred to in reviewer reports. Here, an in-depth analysis of these reports might provide further insights into how the evaluators perceive the allocation of public money to teams with industry engagement. Funding organizations (or the organizations managing the funding process on their behalf) need to develop guidelines to inform evaluators of the potential benefits and costs of industry engagement and, thus, ensure, the effective allocation of public funds so as to optimize U–I collaboration to the benefit of both the organizations and society as a whole. This objective should be made more explicit in calls for project proposals and in evaluator guidelines and on their evaluation forms as interorganizational collaboration can only enhance the frontiers of knowledge and technology.

## Data Availability

The dataset used in this article is not publicly available since the data is confidential. Authorization from the data provider (European Science Foundation) is required for the access. Inquiries concerning the access to the datasets could be directed to Patrick Llerena (pllerena@unistra.fr), the principal investigator of the project GIGA.

## Footnotes

1 For more information, please see http://archives.esf.org/coordinating-research/eurocores/the-scheme.html (Last accessed on 21/06/2021).

2 In this study, the participants of a CRP team are considered as being the PIs of an individual project (IP) within a CRP. CRPs are coordinated by a project leader (PL) and might include self-funding APs, who are also PIs of an IP.

3 Note the Second Stage, carried out by the External Reviewers, was not a selection stage. The reviewers' reports and the scores given to the full proposals acted as a guide to the Expert Panel in the last stage of the process.

4 Each CRP was included within a corresponding program, and an Expert Panel was set up for each program.

5 With the exception of 40 projects, all the projects selected by the Expert Panel in the Third Stage were funded by the respective NFOs.

6 Recall, reviewers were asked to fill out an evaluation form comprising 8–10 sections, each addressing a different criterion. The five specific questions used in our analysis, unlike the others, did not vary over the years and, hence, are used here to establish the average review score.

7 Inverse Mills ratio (IMR) is the ratio of the probability density function to the complementary cumulative distribution function.

8 We were unable to find any common coauthorship relations between the applicant team members and the External Reviewers. This is consistent with the ESF guidelines aimed at avoiding any conflict of interest between the applicant and reviewer. Therefore, the variable *Network proximity* is not included in the Second Stage of analysis.

9 In our analysis, recall, an applicant team refers to a team of leaders comprising PIs. These teams are coordinated by a project leader (PL) who is also a PI of an individual project within a CRP. Teams may include self-funding associate partners (APs) from within or outside Europe.

10 We did not control for the panel's *direct* involvement, as there are no private company-affiliated panel members in our database. Likewise, we decided to exclude the reviewers' *direct* involvement because only a very few (20) present private company affiliation.

11 This result is also in line with a previous study undertaken by the authors [Bianchini et al., 2021 (manuscript submitted for publication)], as part of an ANR GIGA project, investigating gender bias in the EUROCORES evaluation process.
